# Vascular Complications Following Transcatheter Aortic Valve Implantation, Using MANTA (Collagen Plug-Based) versus PROSTAR (Suture-Based), from a French Single-Center Retrospective Registry

**DOI:** 10.3390/jcm12206697

**Published:** 2023-10-23

**Authors:** Clément Benic, Pierre Philippe Nicol, Sinda Hannachi, Martine Gilard, Romain Didier, Bahaa Nasr

**Affiliations:** 1 Department of Cardiology, University Hospital of Brest, 29200 Brest, France; pierre-phillipe.nicol@chu-brest.fr (P.P.N.); sinda.hannachi@chu-brest.fr (S.H.); martine.gilard@gmail.com (M.G.); romain.didier@chu-brest.fr (R.D.); 2 Department of Vascular Surgery, University Hospital of Brest, 29200 Brest, France; bahaa.nasr@chu-brest.fr

**Keywords:** TAVI, vascular complications, MANTA, PROSTAR, femoral access

## Abstract

TAVI requires a large-bore arteriotomy. Closure is usually performed by the suture system. Some studies report a vascular complication rate of up to 21%. MANTA is a recently developed percutaneous closure system dedicated to large caliber vessels based on an anchoring system. Early studies report a lower rate of vascular complications with MANTA devices. This single-center retrospective study included all patients who underwent femoral TAVI at the Brest University Hospital from 20 November 2019 to 31 March 2021. The primary endpoint is the rate of vascular complications (major and minor) pre and post-TAVI procedure. In total, 264 patients were included. There were no significant differences in vascular complications (major and minor) between the two groups (13.6% in the MANTA group versus 21.2% in the PROSTAR group; *p* = 0.105), although there was a tendency to have fewer minor vascular complications in the Manta group (12.1% versus 20.5%; *p* = 0.067). Manta was associated with a lower rate of bleeding complications (3.8% versus 15.2%; *p* = 0.002), predominantly minor complications with fewer closure failures (4.5% versus 13.6%; *p* = 0.01), less use of covered stents (4.5% versus 12.9%; *p* = 0.016), and with no difference in the need for vascular surgery compared to the Prostar group (1.5% versus 2.3%; *p* = 0.652). On the other hand, Manta was associated with a higher rate of femoral stenosis (4.5% versus 0%; *p* = 0.013) without clinical significance (1.5% versus 0%; *p* = 0.156). The Manta and Prostar devices are equivalent in terms of vascular complications. The Manta, compared to the Prostar, is associated with fewer bleeding complications.

## 1. Introduction

Aortic stenosis is the most common valve disease requiring intervention in Europe and the United States, and it increases in frequency in elderly populations, reaching 3–9% of the population after 80 years of age [1]. Severe aortic stenosis has a poor prognosis [2], and aortic valve intervention is strongly recommended, mainly by Transcatheter Aortic Valve Implantation (TAVI) [3].

Most often, TAVI is implanted via the femoral access. The procedure requires the use of a large-caliber approach. Two approaches are classically accepted for the closure of the femoral artery: surgical closure and percutaneous closure with a suture system. However, surgical closure is associated with a longer procedure time, greater blood loss, increased patient discomfort, more sustained anesthesia, increased risk of complications, particularly infectious ones, and slowed down patient ambulation [4].

In the percutaneous approach, the closure of the access requires dedicated closure systems. The majority of these closure devices are suture systems (PROGLIDE^®^ Abbot Vascular, Santa Clara, CA, USA; PROSTAR^®^ Abbot Vascular Abbot Park, IL, USA). With these systems, the rate of minor vascular complications varies from 0 to 18.2%, while major complications vary from 0 to 12.2% [5,6,7,8,9,10,11,12]. Recent studies suggest that suture closure systems have a vascular complication rate of up to 21% [9,11,13,14,15].

The majority of the complications of the approach are related to the use of the closure device [16], affect the short and long-term prognosis, and increase the length of stay as well as the cost of hospitalization [17,18].

Recently, an anchor and collagen closure system dedicated to large vessels has been developed: the MANTA^®^ system (Teleflex, Wayne, PA, USA). The first studies performed with this device showed a low rate of major vascular complications between 0 and 20.9% (Table 1) and, therefore, seems to be an interesting alternative to suture systems for the closure of the femoral approach during a TAVI. There are few data in the literature comparing the Manta to suture systems, including the Prostar closure system in TAVI patients. We, therefore, propose to compare the vascular complications of the MANTA system with the PROSTAR system in patients who have undergone femoral TAVI at the Brest University Hospital.

## 2. Materials and Methods

### 2.1. Study Design and Population

This is a retrospective single-center cohort study including all consecutive patients who underwent femoral TAVI at the Brest University Hospital from 20 November 2019 to 30 March 2021. All patients with severe aortic stenosis and/or severe aortic insufficiency who underwent femoral TAVI were included. The decision to perform TAVI was evaluated by a heart team according to the recommendations of the European Society of Cardiology (ESC). Patients who underwent a hybrid procedure with vascular surgery during TAVI, patients under protective safeguard, not affiliated with social security, or unable to give consent were excluded from the present study. Patients included from 20 November 2019 to September 2020 received suture system (Prostar) closure, whereas patients included from September 2020 to March 2021 received Manta closure. Our center decided to change the closing device in September 2020 for several reasons: the Manta device seemed better suited to calcified lesions, no preclosing time was required, and the device was easy to use.

### 2.2. Analysis of the Femoral Access

**Figure 1:**
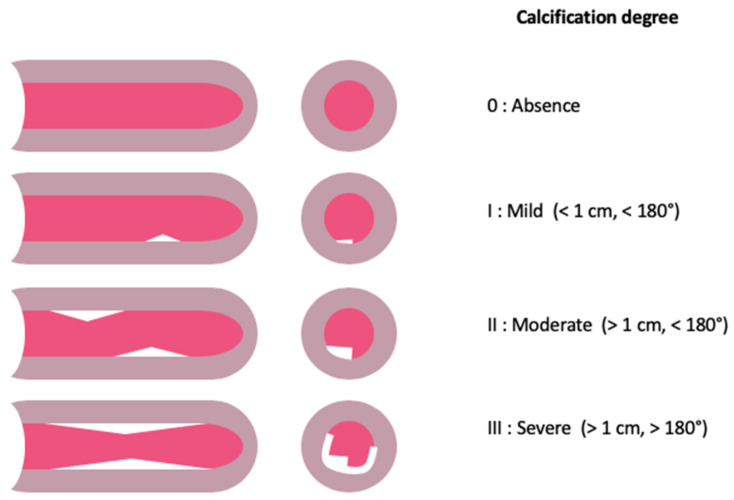
Calcification degree of femoral access.

Femoral analyses were performed by preprocedural CT scan. The characteristics of the main access were studied:

- Diameter of the common femoral artery (mm);
- Degree of calcification (Figure 1);
- Location of calcifications (anterior, medial, lateral, posterior);
- Tortuosity (minimal, moderate, severe);
- Bifurcation height;
- Size of the femoral shaft (external diameter, mm).

The secondary approach was also studied:

- Location.
- Size of the shaft.

### 2.3. Management of Antithrombotic Treatment

All patients were placed on acetylsalicylic acid the day before the TAVI procedure if they were not already receiving long-term treatment. If the patient was already on acetylsalicylic acid, the treatment was continued. If the patient was previously on Clopidogrel or Ticagrelor, the treatment was stopped 5 days before. If the patient was on Direct Oral Anticoagulant (DOA), treatment was stopped 2 days before, while if the patient was on Anti Vitamin K (AVK), treatment was stopped 5 days before with an INR target of 1.5 or less for the procedure. During the procedure, we used unfractionated heparin (UFH) weight-adjusted, and the reversal of anticoagulation was obtained by protamine at the end of the procedure.

### 2.4. TAVI Procedure

The TAVI procedure was performed in the presence of two interventional cardiologists, a radiology manipulator, and two nurses under local. During the use of the Prostar system (from November 2019 to September 2020), for organizational reasons, a cardiothoracic and vascular surgeon was present for the closure of the approach. Arterial puncture of the common femoral artery was performed under fluoroscopy after locating the puncture site by pigtail angiographic opacification of the primary approach (via the secondary approach).

### 2.5. PROSTAR Closure

**Figure 2:**
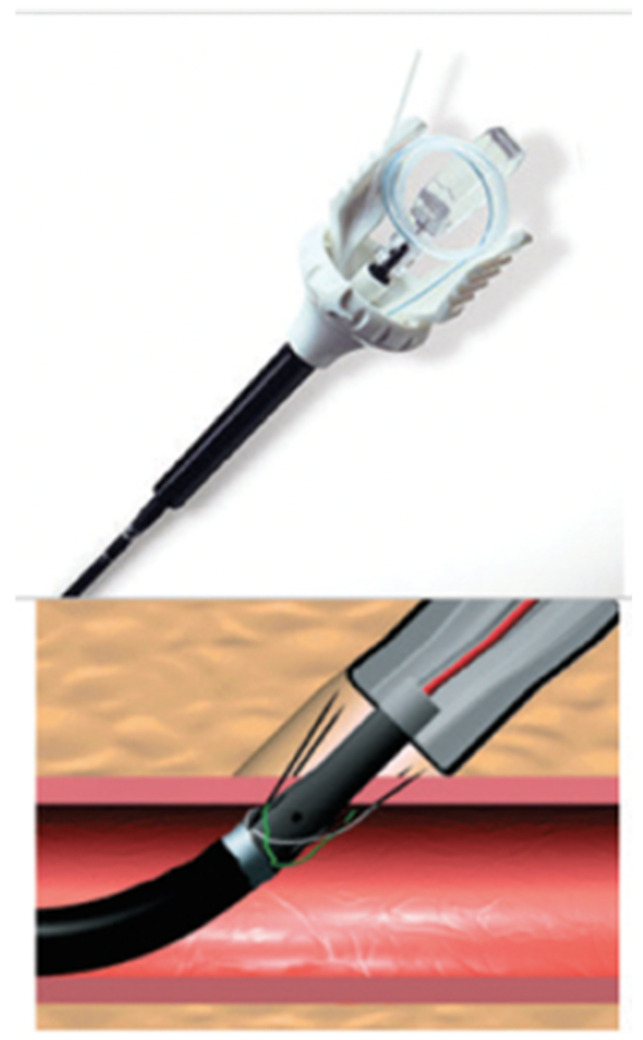
Prostar System: Visualization of the 4 nitinol needles in the vessel wall connected in pairs to a suture.

The Prostar system (Prostar Abbot Vascular Abbot Park, IL, USA-Figure 2) consists of two braided polyester sutures and 4 nitinol needles. This is a pre-closure system; it is placed before the artery is dilated and the procedure is performed. The system deploys the four nitinol needles through the femoral arterial wall, connected two by two to a suture. At the end of the procedure, the polyester sutures are taken up and tightened to close the approach. The system is dedicated to vascular access from 10 to 24 French.

### 2.6. MANTA Closure

**Figure 3:**
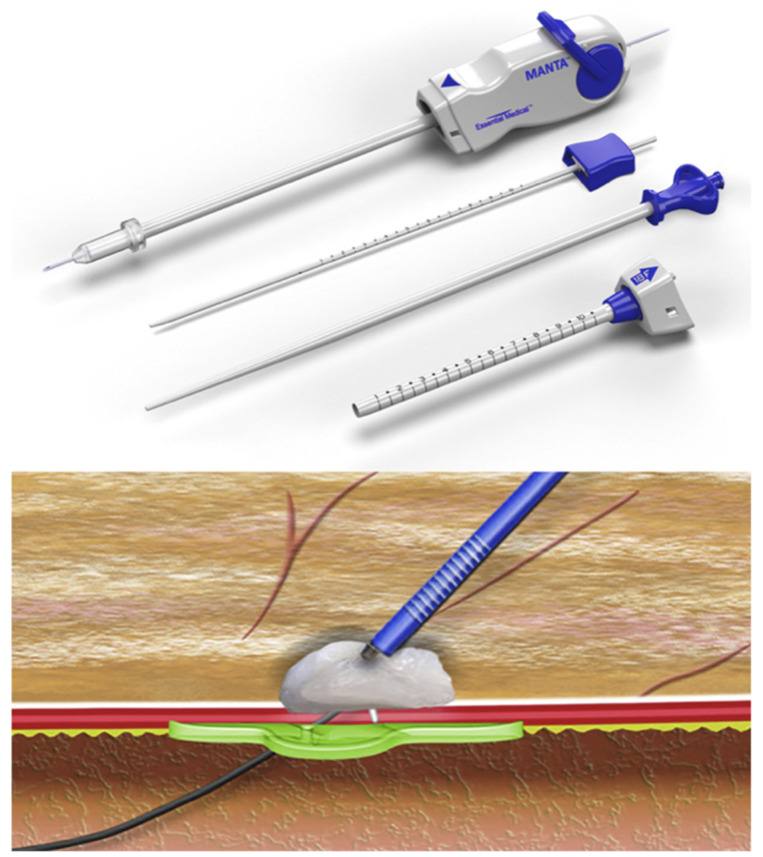
Manta System: Visualization of the bioresorbable part on the inner side of the vessel (green) and the hemostatic collagen (white) on the external side.

The Manta (Teleflex, Wayne, PA, USA—Figure 3) is a closure system dedicated to large-caliber vessels (typically the common femoral artery, 12 to 25 Fr) comprising two parts: a bioresorbable part inside the vessel (made of poly-lactic-co-glycolic-acid) and a hemostatic part made of bovine collagen outside the vessel. The two elements are connected by a non-absorbable polyester suture system. After having located the depth of the femoral artery at the beginning of the procedure with the localization system (8 Fr), at the end of the procedure, the system is advanced on the guide (up to the previously located depth + 1 cm), and then the system is released: the bioresorbable part is plated on the internal face of the vessel. In contrast, the collagen is plated on the external part, allowing the closure of the approach. The components are classically resorbed in 6 months.

### 2.7. Monitoring Procedures after Closure of the Approach

Systematic angiographic monitoring of the main approach was performed immediately after closure. In the absence of abnormalities, a pressure bandage was put in place at the end of the procedure as a complement for 24 h. After the removal of the pressure bandage, a daily clinical examination was performed to look for a local complication. If a complication was identified, vascular Doppler ultrasound was performed as a first step, possibly coupled with a CT scan if necessary.

### 2.8. Primary Endpoint

The primary endpoint was the comparison of total vascular complications (major and minor) between the Manta and Prostar devices at 30 days. Major and minor vascular complications were defined according to the Valve Academic Research Consortium-2 (VARC-2) classification.

### 2.9. Secondary Endpoints

The secondary endpoints were the comparison between the two groups:

- In-hospital mortality.
- Type of vascular complications: false aneurysm, arteriovenous fistula arterial dissection, arterial stenosis (>50% reduction in diameter), homolateral lower limb ischemia, bleeding.
- Bleeding complications: Total, major (defined as loss of more than 3 g/dL of hemoglobin, the need to transfuse more than 2 red blood cells or the need for surgery), and minor (any clinically significant non-life-threatening bleeding that does not meet the criteria for major bleeding).
- Immediate failure of closure of the approach by the device—defined by the need for additional endovascular or surgical closure per procedure.
- Length of stay.
- Study of the risk factors for complications of the approach.

### 2.10. Ethics

Consent was obtained for each patient. The protocol was carried out in accordance with the Declaration of Helsinki and the rules of good clinical practice.

### 2.11. Statistics

Continuous variables are presented with their mean and standard deviation. Categorical variables are presented in absolute (n) and relative (%) values. To compare two quantitative variables, a Student’s *t* test was performed. For the comparison of two qualitative variables, a Chi-2 test was performed. For factors favoring vascular complications, a univariate and then multivariate analysis was performed. For the multivariate analysis, only the values for which *p* was less than 0.05 in the univariate analysis were selected. Redundant data were not selected for the multivariate analysis. A total number of variables not exceeding 10% of the total number of events could be used. Multivariate analysis was performed by multivariate logistic regression. Statistical tests were performed with IBM SPSS Statistics version 25.0, Chicago, IL, USA. A difference is considered significant if a two-sided *p* was <0.05.

## 3. Results

### 3.1. Flow Chart

**Figure 4:**
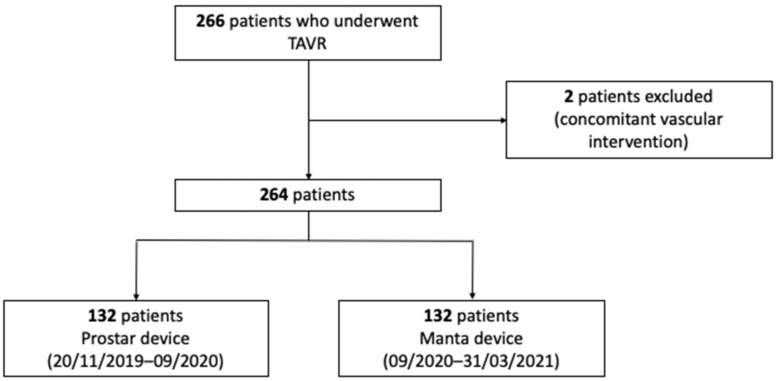
Flow chart. (TAVR: TransAortic Valve Replacement).

Over the period 20 November 2019 to 31 March 2021, 266 patients were eligible. Two patients were excluded because of scheduled vascular surgery concomitant with TAVI. A total of 264 patients were therefore included: 132 patients in the Manta group and 132 patients in the Prostar group (Figure 4).

### 3.2. Characteristics of Study Population

Characteristics of study population are summarized in Table 2. The mean age of the included patients was 82.6 years. The cardiovascular risk factors of the patients were hypertension in 77.3%, dyslipidemia in 53.4%, smoking (cessation or active) in 31.1%, and diabetes in 17.4%. Patients had a history of coronary angioplasty in 19.3% of cases and a PAD in 6.8% of cases. A minority of the patients had undergone previous cardiac surgery: coronary artery bypass grafting in 6.4% of cases and aortic valve replacement by bioprosthesis in 1.9% of cases. Thirty-one percent of the patients were on long-term curative anticoagulation (AOD or VKA).

### 3.3. Characteristics of the Femoral Access and Implanted Valve

The characteristics of femoral access are summarized in Table 3. The main access was more frequently performed via the right femoral artery (75% versus 25% via the left femoral approach). The mean diameter of the main approach was 7.64 mm, with a slightly larger diameter in the Prostar group (7.8 +/− 1.1 mm in the Prostar group versus 7.5 +/− 1 mm in the Manta group; *p* = 0.038). The mean ratio of sheath to femoral artery size was 0.83 and was comparable between the two groups (*p* = 0.098). Calcifications were most often minimal to moderate (75% of cases) and were most often located posteriorly and medially (71.6% and 46.6%, respectively). The femoral bifurcation was high in 9.1% of cases, and iliofemoral tortuosity was most often minimal to moderate (84.1%). The radial approach was the preferred secondary approach (62.9% of cases). The main valve used was the Sapiens 3 valve in 60.7% of cases.

### 3.4. Vascular Complications of the Main Access

**Figure 5:**
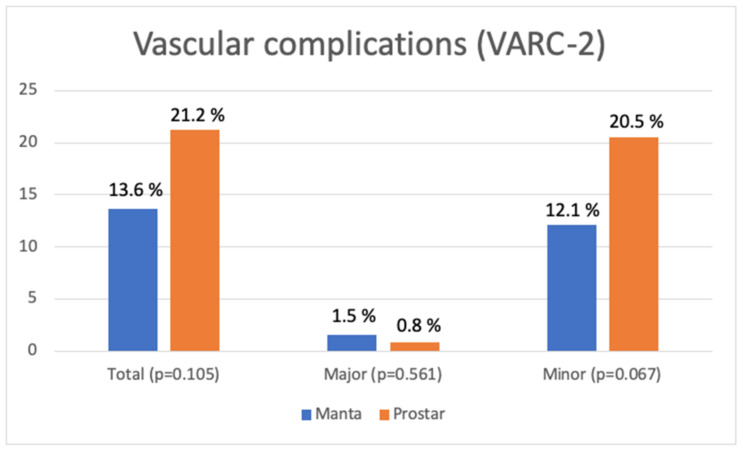
Vascular complications (VARC-2) according to the closing device.

There were no differences in terms of vascular complications of the main approach between the two devices, either for total, major, or minor vascular complications (Table 4 and Figure 5). However, there was a non-significant trend towards a lower rate of minor complications in the Manta group (12.1% versus 20.5% in the Prostar group, *p* = 0.067).

**Figure 6:**
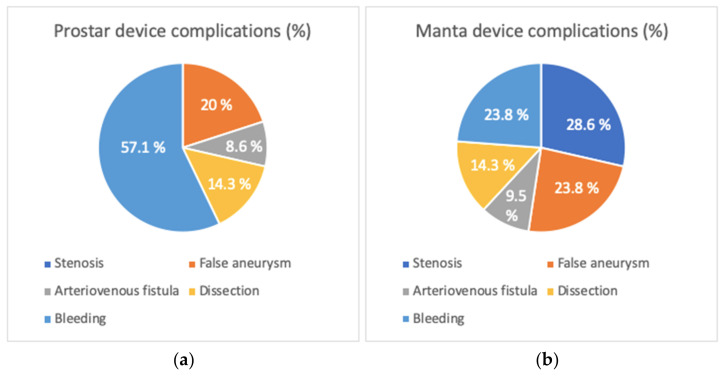
Type and distribution of vascular complications for (**a**) PROSTAR device and (**b**) MANTA device.

When we look precisely at vascular complications (Figure 6), there was a higher rate of femoral artery stenosis in the Manta group compared to the Prostar group (4.5% versus 0%; *p* = 0.013), but without difference in the rate of homolateral lower limb ischemia (*p* = 0.156). There were no differences between the two devices for false aneurysms (*p* = 0.555), arteriovenous fistulas (AVF) (*p* = 0.652), and arterial dissections (*p* = 0.473).

**Figure 7:**
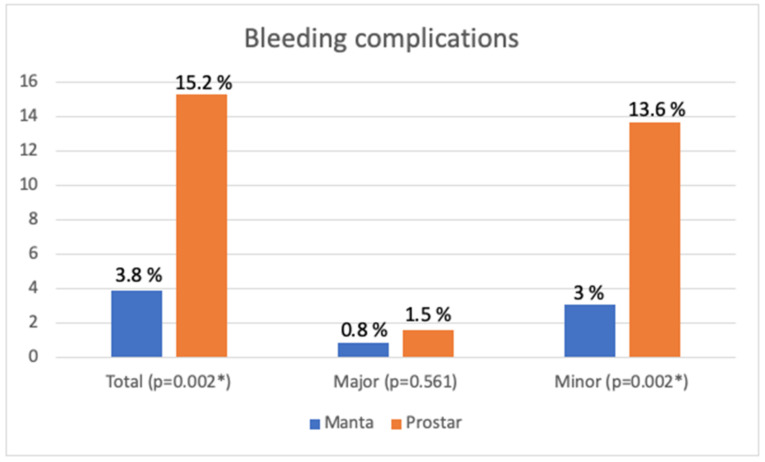
Bleeding complications according to the closing device (* *p* < 0.05).

Failure to close was more frequent with the Prostar device (13.6% versus 4.5%; *p* = 0.01). Access bleeding (Figure 7) was more frequent with the Prostar device (15.2% versus 3.8% with the Manta system; *p* = 0.002), mainly due to minor bleeding (*p* = 0.002), while there was no significant difference between the groups for major bleeding (*p* = 0.561).

The use of a covered stent was more frequent with the Prostar system (12.9% versus 4.5% with the Manta system; *p* = 0.016), while the use of vascular surgery was not significantly different between the two groups (*p* = 0.652). There was a non-significant trend (*p* = 0.053) towards a higher transfusion rate in the Prostar group (6% versus 1.5% in the Manta group). There was no difference in the amount of contrast medium used between the two groups (*p* = 0.537).

### 3.5. In-Hospital Mortality and Length of Stay

Intra-hospital mortality related to the TAVI procedure remains relatively rare in this registry, with 0.4% of cases in total. Only one death was observed and was related to a complication of the approach to a false aneurysm with multiple surgical revisions in the Prostar group. There were no significant differences in in-hospital mortality between the two groups (*p* = 0.316).

Post-implantation length of stay was significantly shorter in the Manta group (4.9 +/− 3.9 days versus 7.0 +/− 5.7 days in the Prostar group, *p* = 0.001).

### 3.6. Risk Factors for Vascular Complications

Factors associated with a risk of developing a vascular complication are presented in Table 5. In univariate analysis, variables associated with risk of developing a vascular complication were diabetes (30.4% versus 14.7%; *p* = 0.01), the ratio sheath to the size of the common femoral artery (87.5 +/− 11.5% versus 81.1 +/− 13.7%; *p* = 0.003), Euroscore Logistic (9.53 +/− 1.41 versus 6.33 +/− 0.43; *p* = 0.043), creatinine level (112.37 +/− 91.94 μmol/L versus 90.33 +/− 42.84 μmol/L; *p* = 0.013), and common femoral artery diameter (7.26 +/− 1.09 mm versus 7.72 +/− 1.07 mm; *p* = 0.009).

Age, gender, BMI, HBP, degree of calcification, location of calcification, high bifurcation, PAD, platelet count, GFR, and previous anticoagulant therapy were not found to be significantly associated with the risk of developing a vascular complication.

In multivariate analysis, variables associated with the risk of developing a vascular complication were diabetes (OR 2.49, CI 95 1.16–5.3, *p* = 0.019), sheath/femoral ratio (OR = 1.045, CI 95 1.02–1.07, *p* = 0.002), and Logistic Euroscore (OR = 1.046, CI 95 1.002–1.092, *p* = 0.042).

However, creatinine level was not found to be significant in multivariate analysis (OR = 1.005, CI 95 1–1.010, *p* = 0.046).

## 4. Discussion

In this single-center retrospective study including 264 consecutive patients (132 patients per group), the main findings were: (1) No significant difference in terms of vascular complications (major and minor) between the two closure devices was found, while there was a trend towards a lower rate of minor vascular complications in the Manta group (*p* = 0.067), although this did not reach significance. (2) A higher rate of bleeding complications was observed with the Prostar device compared to the Manta, predominantly in minor bleeding. (3) A higher rate of femoral stenosis after closure was found in the Manta group, although there were no clinically significant differences between the two groups in terms of homolateral lower limb ischemia.

### 4.1. Vascular Complications

The data from this study are consistent with the literature in terms of vascular complications, with no significant differences between MANTA and suture-based devices [9,10,11,12,25,29,30,31,32].

In terms of specific complications, there was a higher rate of femoral stenosis in the Manta group, with no difference in terms of clinical impact or need for surgery. Moccetti et al. [21] report this same difference, although this was not found in the most recent meta-analysis [29,30,31,32]. Femoral stenosis can be due to poor toggle positioning of the anchor on the internal side of the artery, especially in the presence of calcifications and in small femoral arteries, and this could be an explanation for the higher rate of femoral stenosis in our study.

### 4.2. Bleeding Complications

There is a higher rate of bleeding complications in the Prostar group compared to the Manta system in our study, mainly driven by minor bleeding. These results are in line with the Moriyama study [9], where the bleeding complication rate was 18% in the MANTA group versus 33% in the ProGlide group (OR = 0.44, CI 95 0.23–0.83, *p* = 0.01). However, this difference was not found in the most recent meta-analysis in terms of major and minor bleeding complications [29,30,31,32].

We can speculate that this higher rate of bleeding complications in our study was related to higher immediate closure failure in the Prostar group (13.6% versus 4.5% in the Manta group, *p* = 0.01), which is in line with the literature with suture-based devices [29,30,31,32] and required more frequent use of covered stents.

### 4.3. Independent Risk Factors of Vascular Complication

The three independent risk factors of vascular complication were found in our study:

- Ratio-sheath to the femoral, which is consistent with the current literature [33,34].
- Euroscore logistic is representative of the patient’s severity.
- Finally, diabetes mellitus, which was also a risk factor for false aneurysm in the site of femoral access [35]

In our study, age, gender, BMI, HBP, renal insufficiency, and the degree and location of calcification were not identified as risk factors for vascular complication, which is in line with other studies [9,12,24]. However, some studies highlighted a link between vascular complications and gender, HBP, renal insufficiency, PAD, and the degree of calcification [9,21,23].

### 4.4. Limits and Perspectives

This study has several limitations; firstly, it is a single-center retrospective cohort study, with the usual limitations. The length of stay was shorter in our study in the Manta group, but we cannot exclude a time effect. No comparison with the PROGLIDE device was performed in the present study since it is not routinely used in our center.

Therefore, further prospective multicenter randomized controlled studies comparing the Manta with the suture system for closure of the main approach during a TAVI procedure are needed to confirm these results. The use of vascular ultrasound to guide the puncture could be an additional aid to decrease the overall rate of vascular complications, as suggested in some studies [36,37].

## 5. Conclusions

There were no differences in the rates of vascular complications between the Manta and Prostar devices. However, there was a higher rate of bleeding complications in the Prostar group as compared to the manta group, mainly driven by the rate of minor bleeding in the Prostar group and requiring more frequent use of a covered stent. In contrast, the Manta is associated with more non-clinically significant vascular femoral stenosis.

## Figures and Tables

**Table 1:** Systematic review about vascular complications Manta device in TAVI patients. (HBP: High Blood Pressure, PAD: Peripheral Artery Disease, GFR: Glomerular Filtration Rate).

	Vascular Complications (Total/Major/Minor)	Other Closing Devices and Vascular Complications	Risk Factors for Vascular Complications
Van Mieghem et al., 2017 [19] (n = 50)	**Manta** (n = 50) Total 2% Major 2% Minor 0%		
Biancari et al., 2018 [10] (n = 222)	**Manta** (n = 107) Total 13% Major 9.3% Minor 3.7%	**Proglide** (n = 115) Total 14.8% Major 12.2% Minor 2.6%	
Hoffman et al., 2018 [7] (n = 151)	**Manta** (n =75) Major 10.7%	**Proglide** (n = 76) Major 2.7%	
De Palma et al., 2018 [8] (n = 346)	**Manta** (n = 107) Major and death 1.1%	**Prostar XL** (n = 239) Major and death 1.9%	
Moriyama et al., 2019 [9] (n = 222)	**Manta** (n = 111) Total 14% Major 7% Minor 6%	**Proglide** (n = 111) Total 21% Major 8% Minor 13%	- **Glomerular filtration rate (GFR)** (OR 0.63, CI 95 0.21–0.95; *p* = 0.04)
Wood et al. the SAFE MANTA Study, 2019 [20] (n = 263)	**Manta** (n = 263) Major 4.2%		
Gheorghe et al., 2019 [11] (n = 366)	**Manta** (n = 168) Total 11.3% Major 0.6% Minor 10.7%	**Prostar XL** (n = 198) Total 19.7% Major 1.5% Minor 18.2%	
Moccetti et al., 2019 [21] (n = 100)	**Manta** (n = 100) Total 14% Major 9% Minor 5%		- **Peripheral arterial disease (PAD)** (45.5% versus 13.5%; *p* = 0.008) - **Diameter of the femoral artery** (5.7 ± 1.1 mm versus 7.4 +/− 1.8 mm; *p* = 0.006)
Halim et al., 2020 [22] (n = 73)	**Manta** (n = 73) Total 13.7% Major 0% Minor 13.7%		
Kroon et al., the MARVEL Study 2020 [23] (n = 500)	**Manta** (n = 500) Total 9.6% Major 4% Minor 5.6%		- **Severe calcifications** (OR = 2.72, CI 95 1.06–7.03; *p* = 0.038) - **Scarring of the approach due to previous procedure** (OR 16.55 CI 95 2.72–100.59; *p* = 0.002) - **Length of procedure** (0R 1.04 CI 95 1.02–1.05; *p* = 0.0005) - **Female** (OR 2.13, CI 95 1.05–4.34; *p* = 0.037) - **High blood pressure (HBP)** (OR 2.82 CI 95 1.14–6.97; *p* = 0.025)
Van Wiechen et al., the MASH study, 2021 [12] (n = 206)	**Manta** (n = 102) Total 10% Major 2% Minor 8%	**Proglide** (n = 104) Total 4% Major 0% Minor 4%	
Van Wiechen et al., 2021 [24] (n = 512)	**Manta** (n = 512) Total 8% Major 4% Minor 4%		- **Diameter of the femoral artery** (OR 0.70 CI 95 0.53–0.93; *p* = 0.01) - **Low arterial puncture** (OR 3.47 CI 95 1.21–10; *p* = 0.02) and high (OR 2.43 IC 95 1.16.–5.10; *p* = 0.02)
Ali et al., 2021 [25] (n = 136)	**Manta** (n = 50) Total 10% Major 0% Minor 10%	**Proglide** (n = 86) Total 10.5% Major 3.5% Minor 7%	
Dumpies et al., 2021 [26] (n = 578)	**Manta** (n = 195) Total 10.7% Major 2% Minor 8.7%	**Proglide** (n = 383) Total 19% Major 6.5% Minor 12.5%	
Sarathy et al., 2021 [27] (n = 132)	**Manta** (n = 86) Total 18% Major 6% Minor 12%	**Proglide** (n = 86) Total 13% Major 6% Minor 7%	
Abdel Wahab et al., 2022 CHOICE-CLOSURE [28] (n = 516)	**Manta** (n = 258) Total 20.9% Major 5% Minor 15.9%	**Proglide** (n = 258) Total 14.7% Major 1.9% Minor 12.8%	

**Table 2:** Characteristics of the study population (*: *p* < 0.05). (BMI: Body Mass Index, HBP: High Blood Pressure, PAD: Peripheral Artery Disease, GFR: Glomerular Filtration Rate).

	Total (n = 264)	Manta (n = 132)	Prostar (n = 132)	*p*
Age	82.6 +/− 0.5	82.6 +/− 5.4	82.6 +/− 5.5	0.973
Gender% Male/Female	57/43 (151/113)	54.5/45.5 (72/60)	59.8/41.2 (79/53)	0.384
Body Mass Index (BMI)	26.0 +/− 4.3	26.12 +/− 4.16	25.90 +/− 4.45	0.674
Euroscore logistic	8.8 +/− 7	8.61 +/− 7.13	8.99 +/− 6.93	0.659
HBP	77.3% (204/264)	74.2% (98/132)	80.3% (106/132)	0.240
Dyslipidemia	53.4% (141/264)	53% (70/132)	53.8% (71/132)	0.902
Smoking No Active Cessation	68.9% (182/264) 1.9% (5/264) 29.2% (77/264)	65.9% (87/132) 2.3% (3/132) 31.8% (42/132)	72.0% (95/132) 1.5% (2/132) 26.5% (35/132)	0.552
Diabetes	17.4% (46/264)	15.1% (20/132)	19.7% (26/132)	0.330
Stroke	7.2% (19/264)	9% (12/132)	5.3% (7/132)	0.234
Previous coronary bypass surgery	6.4% (17/264)	6% (8/132)	6.8% (9/132)	0.802
Aortic valve bioprosthesis	1.9% (5/264)	1.5% (2/132)	2.3% (3/132)	0.652
Previous coronary stenting	19.3% (51/264)	22.7% (30/132)	15.9% (21/132)	0.161
PAD	6.8% (18/264)	6.8% (9/132)	6.8% (9/132)	0.802
Pacemaker	9.1% (24/264)	10.6% (14/132)	7.6% (10/132)	0.392
Atrial fibrillation	15.1% (40/264)	17.4% (23/132)	14.4% (19/132)	0.501
Anticoagulation	31% (82/264)	25.7% (34/132)	36.3% (48/132)	0.063
Hemoglobin (g/dL)	12.4 +/− 1.4	12.36 +/− 1.41	12.39 +/− 1.47	0.878
Platelets (G/L)	221 +/− 69	230 +/− 77	213 +/− 60	0.046 *
GFR (mL/mn)	57.3 +/− 22.8	55.84 +/− 21.31	58.75 +/− 24.20	0.302

**Table 3:** Femoral access characteristics and type of implanted valve (*: *p* < 0.05).

	Total (n = 264)	Manta (n = 132)	Prostar (n = 132)	*p*
Laterality Right Left	75% 25%	67.4% (89/132) 32.5% (43/132)	81.8% (108/132) 18.2% (24/132)	0.007 *
Diameter (mm)	7.64 +/− 1.07	7.5 +/− 1.0	7.8 +/− 1.1	0.038 *
Sheath/femoral ratio	0.83 +/−0.11	0.84 +/− 0.11	0.82 +/− 0.12	0.098
Calcifications 0 I II III	12.5% (33/264) 37.9% (100/264) 37.1% (98/264) 12.5% (33/264)	10.6% (14/132) 41.7% (55/132) 40.2% (53/132) 7.7% (10/132)	14.4% (19/132) 34.1% (45/132) 34.1% (45/132) 17.4% (23/132)	0.057
Calcification location Anterior Medial Lateral Posterior	12.5% (33/264) 46.6% (123/264) 11.7% (31/264) 71.6% (189/264)	12.9% (17/132) 46.2% (61/132) 11.4% (15/132) 67.4% (89/132)	12.1% (16/132) 46.9% (62/132) 12.1% (16/132) 75.8% (100/132)	0.852 0.902 0.848 0.133
High bifurcation	9.1% (24/264)	8.3% (11/132)	9.8% (13/132)	0.669
Tortuosity Mild Moderate Severe	39.4% (104/264) 44.7% (118/264) 15.5% (41/264)	40.9% (54/132) 47% (62/132) 12.1% (16/132)	37.9% (50/132) 42.4% (56/132) 18.9% (25/132)	0.297
Valve implanted Acurate Neo Corevalve Sapiens Portico	9.1% (24/264) 28.8% (76/264) 60.7% (159/264) 1.1% (3/264)	11.3% (15/132) 29.5% (39/132) 55.3% (73/132) 2.3% (3/132)	6.8% (9/132) 28% (37/132) 65.1% (86/132) 0% (0/132)	0.133
Sheath size (mm)	6.2 +/− 0.32	6.2 +/− 0.32	6.2 +/− 0.33	0.277
Secondary access Radial Femoral Humeral	62.9% (176/264) 36.0% (95/264) 1.1% (3/264)	67.4% (89/132) 31.1% (41/132) 1.5% (2/132)	58.3% (77/132) 40.9% (54/132) 0.8% (1/132)	0.225

**Table 4:** Complication rates by device and need for therapy (* *p* < 0.05).

	Total (n =264)	Manta (n = 132)	Prostar (n = 132)	*p*
Death	0.4% (1/264)	0% (0/132)	0.8% (1/132)	0.316
Vascular complications Total Major Minor	17.4% (46/264) 1.1% (3/264) 16.3% (43/264)	13.6% (18/132) 1.5% (2/132) 12.1% (16/132)	21.2% (28/132) 0.8% (1/132) 20.5% (27/132)	0.105 0.561 0.067
Stenosis	2.3% (6/264)	4.5% (6/132)	0% (0/132)	0.013 *
Lower limb ischemia	0.8% (2/264)	1.5% (2/132)	0% (0/132)	0.156
False aneurysm	4.5% (12/264)	3.8% (5/132)	5.3% (7/132)	0.555
Arteriovenous fistula	1.9% (5/264)	1.5%(2/132)	2.3% (3/132)	0.652
Dissection	3% (8/264)	2.3% (3/132)	3.8% (5/132)	0.473
Closure failure	9.1% (24/264)	4.5% (6/132)	13.6% (18/132)	0.01 *
Bleeding Total Major Minor	9.5% (25/264) 1.1% (3/264) 8.3% (22/264)	3.8% (5/132) 0.8% (1/132) 3% (4/132)	15.2% (20/132) 1.5% (2/132) 13.6% (18/132)	0.002 * 0.561 0.002 *
Covered stent	8.7% (23/264)	4.5% (6/132)	12.9% (17/132)	0.016 *
Vascular surgery	1.9% (5/264)	1.5% (2/132)	2.3% (3/132)	0.652
Transfusion	3.8% (10/264)	1.5% (2/132)	6% (8/132)	0.053
Length of stay (days)	5.95 +/− 5.0	4.9 +/− 3.9	7.0 +/− 5.7	0.001 *
Amount contrast medium (mL)	76.2 +/− 28.5	77.3 +/− 27.6	75.1 +/− 29.5	0.537

**Table 5:** Univariate and multivariate analysis of risk factors for vascular complications (*: *p* < 0.05).

Univariate Analysis				Multivariate Analysis		
	**Complications** **(n = 46)**	**No Complication** **(n = 218)**	***p***	**OR**	**IC 95**	***p***
Diabetes	30.4% (14/46)	14.7% (32/218)	0.01 *	2.49	1.16–5.3	0.019 *
Sheath/femoral ratio (%)	87.5 +/− 11.5	81.1 +/− 13.7	0.003 *	1.045	1.02–1.07	0.002 *
Euroscore logistic	9.53 +/− 1.41	6.33 +/− 0.43	0.043 *	1.046	1.002–1.092	0.042 *
Creatinine (micromoles/L)	112.37 +/− 91.94	90.33 +/− 42.84	0.013 *	1.005	1–1.010	0.046
Diameter of the common femoral artery (mm)	7.26 +/− 1.09	7.72 +/− 1.07	0.009 *			

## Data Availability

The data will be no available online.
